# Arrhythmias After Heart Transplantation: Mechanisms and Management

**DOI:** 10.1161/JAHA.112.001461

**Published:** 2012-04-24

**Authors:** Anees Thajudeen, Eric C. Stecker, Michael Shehata, Jignesh Patel, Xunzhang Wang, John H. McAnulty, Jon Kobashigawa, Sumeet S. Chugh

**Affiliations:** Cedars-Sinai Heart Institute, Los Angeles, CA (A.T., M.S., J.P., X.W., J.K., S.S.C.); Oregon Health and Science University, Portland, OR (E.S.); Legacy Good Samaritan Medical CenterPortland, OR (J.H.M.)

**Keywords:** arrhythmia (heart rhythm disorders), arrhythmia (mechanisms), transplantation, atrial fibrillation

## Introduction

Heart transplantation (HT) has significantly altered the treatment paradigm for end-stage heart disease. With current surgical techniques and postoperative immunosuppression, 1-year survival after HT is ≈90%, 5-year survival is ≈70%, and median survival exceeds 10 years.^1–3^

These improved outcomes have also affected the natural history of arrhythmia occurrence in the HT patient, and arrhythmias are increasingly recognized as significantly affecting quality of life, morbidity, and survival. Besides the effects of surgical healing, the increasing longevity of the HT patient allows for new and progressive alterations in the donor heart as well as the neurohumoral milieu, resulting in a spectrum of arrhythmias with clinical implications. The Table provides a summary of the unique mechanisms of arrhythmias in the transplanted heart.

**Table:** Major Mechanisms of and Considerations for Arrhythmias After Heart Transplantation

Arrhythmia	Mechanisms	Considerations
Sinus bradycardia	• Denervation	• Common
	• Sinus node ischemia/injury	• Usually reversible
	• Tachycardia-bradycardia syndrome	• PPI if symptomatic and irreversible
	• Drug induced	

Conduction system disease	• RBBB	• Isolated RBBB probably has no prognostic significance
	•Graft ischemia/RV injury	
	•Unknown mechanism	
	• Progressive conduction disease	• Associated with worse prognosis
	• Injury due to EMB	
	•Cardiac allograft vasculopathy	
	•Chronic rejection	
	•Ventricular dysfunction/remodeling	

Atrial fibrillation	• Postoperative	• Low incidence in postoperative period compared to major cardiac surgery
	•Ischemia	• Lone atrial fibrillation or pulmonary vein triggers unlikely
	•Denervation	• Secondary cardiac and noncardiac cause to be evaluated
	•Pericardial inflammation	• Potential drug interactions with immunosuppression
	•Autonomic hypersensitivity	
	•Primary graft failure	
	•Early rejection	
	•Inotropes	
	• Late	
	•Ventricular dysfunction	
	• Valvular regurgitation	
	•Rejection	
	•Systemic inflammation	
	•Allograft vasculopathy	
	•Focal trigger from SVC/IVC/CS	

Atrial flutter	• Rejection	• Most common arrhythmia on follow-up
	• Atrial remodeling (same causes as AF)	• Both isthmus-dependent and non–isthmus-dependent mechanisms
	• Atrial suture lines—conduction barriers	• Stable patients amenable to RFA
	• Recipient-to-donor atrial conduction	

Other supraventricular tachycardia	• Recipient-to-donor conduction of sinus beats	• Most forms amenable to RFA
	• Recipient atrial flutter or fibrillation	
	• Focal microreentry	
	• Ectopic tachycardia from donor atria	
	• AVNRT and AVRT	

Nonsustained VT	• Perioperative	• Significance not clear
	• Late	• Evaluate for SCD risk if recurrent or symptomatic
	•Graft vasculopathy	
	•Rejection	

Sustained VT	• LV dysfunction	• May be associated with hyperacute rejection
	• Rejection	• Evaluate for SCD risk
	• Allograft vasculopathy	• Probable indication for ICD

AVNRT indicates AV nodal reentrant tachycardia; AVRT, AV reentrant tachycardia; CS, coronary sinus; EMB, endomyocardial biopsy; ICD, implantable cardioverter defibrillator; IVC, inferior vena cava; LV, left ventricular; PPI, permanent pacemaker implantation; RBBB, right bundle branch block; RFA, radiofrequency ablation; RV, right ventricular; SCD, sudden cardiac death; SVC, superior vena cava; and VT, ventricular tachycardia.

## Mechanisms and Substrates

### Graft Ischemia Time

Prolonged graft ischemia time can predispose to conduction system injury in both early and late postoperative periods. Perioperative ischemic damage and subsequent endocardial fibrosis likely play a mechanistic role in many cases. Patients with prolonged graft ischemia >4 hours are classified as high risk and have greater 30-day and 1-year mortality rates.^4,5^ Risk of chronic rejection secondary to enhanced activation of the graft vessel endothelium may also be increased when myocardial preservation is not adequate.

### Bicaval Versus Biatrial Anastomosis

The most commonly used technique of donor-to-recipient anastomosis is the bicaval method, in which anastomoses are made at the level of the two vena cavae, the great vessels, and the left atrial cuff around the pulmonary veins. Few centers continue to use the original biatrial method described by Shumway, where part of the recipient right and left atria are retained and sutured to the respective atria of the donor. With the latter method, the recipient sinus node is preserved but is not functional because of disruption of blood supply and denervation. Moreover, there is complete conduction block across the suture line in the right atrium. With the bicaval method, there is less sinus nodal injury, tricuspid regurgitation, and atrial dilatation.^6^

When the biatrial method is used, activation of the recipient atrial tissue may be reflected on the ECG. In combination with graft P waves, the native P waves may mimic atrial flutter, though close examination will reveal nonconducted atrial parasystole rather than atrial flutter.^7^ Reestablishment of conduction across the atrial anastomosis may produce tachycardia because of fibrillatory activity or flutter activity in the recipient atrium.^8–12^ Sinus activity from the recipient atrium may intermittently escape into the donor atrium and manifest as frequent atrial ectopics or an atrial parasystole. The scars in the atria act as conduction barriers and can also predispose to atrial flutters—cavo-tricuspid isthmus dependent as well as mitral annular flutters. Thus, the biatrial method is likely associated with greater risk of reentrant tachycardia and flutter (though not supported by all series).^13–15^

The exclusion of the pulmonary veins and the posterior left atrium is thought to be responsible for the very low incidence of atrial fibrillation (AF) with either surgical method, compared with other major cardiac surgeries including bilateral lung transplantation.^14–16^

### Denervation and Reinnervation

The donor heart is completely denervated during transplantation. In the balance, lack of parasympathetic activity has greater effects, and most HT patients have higher than average resting heart rate and significantly reduced heart rate

variability. Over time, both sympathetic and parasympathetic reinnervation will occur, but the degree of reinnervation is incomplete, nonuniform, variable between patients, and heterogeneous within the same patient.^17,18^ Studies have correlated changes in the corrected QT interval to sympathetic reinnervation and have postulated that there may be a subset of patients with increased ventricular arrhythmia and mortality risk associated with heterogeneous reinnervation.^19,20^ Autonomic denervation may partially account for several unique electro-physiological findings in HT patients, beginning with the low incidence of AF after HT. Denervation is also an intriguing possible factor in the lower incidence of ventricular fibrillation (VF) as the terminal rhythm among HT patients who have sudden cardiac death (SCD).^21^ Finally, hypersensitivity to adenosine as a result of denervation is the likely mechanism for exaggerated sinus node and AV node suppression with adenosine after HT.

### Cardiac Allograft Vasculopathy

Cardiac allograft vasculopathy is a relatively common occurrence and an important prognostic indicator late after transplantation.^5,22^ Ischemia resulting from vasculopathy or atherosclerosis is a likely precipitant of ventricular arrhythmias and SCD. Ischemia and infarction can lead to left ventricular (LV) dysfunction, with consequent increased risk of SCD. Progressive LV dysfunction, sustained ventricular arrhythmias, unexplained syncope, and progressive conduction system disease are indications for unscheduled coronary angiography in many transplant programs. Selected patients with severe coronary artery disease and LV dysfunction may receive implantable cardioverter defibrillators (ICDs) with the hope of preventing SCD, although the exact mechanisms and benefits are unclear.

### Nonspecific Late Graft Failure

When progressive LV dysfunction occurs without evidence of epicardial coronary narrowing or evidence of rejection by biopsy, it is labeled as nonspecific graft failure. Although the exact relationship to incident arrhythmias is not clear,^23^ such patients can go on to develop severe LV dysfunction and terminal arrhythmia manifesting as pulseless electrical activity (PEA) and asystole.^23^

### Rejection

Over time, the incidence of rejection has decreased to 20% to 30% in the first posttransplantation year but can occur at any time period after HT.^5^ Many arrhythmias, especially AF and flutter, have been attributed to acute rejection.^24,25^ Although some studies observing high incidence of atrial arrhythmia reported no association with rejection,^26–28^ other studies have shown an association of sustained AF and atrial flutter with rejection episodes.^15,16,25,29^ The occurrence of persistent or paroxysmal AF should prompt evaluation for rejection.^15^ In HT patients, atrial flutter can occur in the setting of rejection^25,28,30^ or can be a manifestation of remodeled and scarred atria that can be associated with cardiac allograft vasculopathy.^15,16^ Repeated rejection episodes may lead to cumulative damage as a mechanism of atrial flutter.^25^ However, no clear relationship has been established between ventricular arrhythmias/SCD and rejection episodes. Myocardial injury due to infiltration of inflammatory cells, edema, and subsequent scarring and ventricular dysfunction may predispose to arrhythmias. Patients with severe acute rejection can have SCD. Routine monitoring of the ECG is not recommended for acute allograft rejection.^31^

## Arrhythmias: Manifestations and Management

### Bradycardia and Conduction System Disease

Sympathetic denervation, ischemic injury to the sinus node, graft ischemia, and drug effects are the common underlying causes of posttransplantation bradycardia.^28,32–34^ Some of these patients have tachycardia-bradycardia syndrome, and drugs for treatment of atrial arrhythmia may worsen the bradycardia.^28^ A potential association of bradycardia with increased likelihood of rejection or graft vasculopathy is controversial. Complete AV block has been reported late after HT, with multiple possible etiologies, including postoperative injury, progressive conduction system disease associated with coronary artery disease, LV dysfunction, chronic rejection, and injury from endomyocardial biopsies.^35–37^ The available retrospective series are not able to provide an exact prevalence of bradycardia episodes and AV block, but risk increases with time after transplantation.^38^

**Figure 1.**
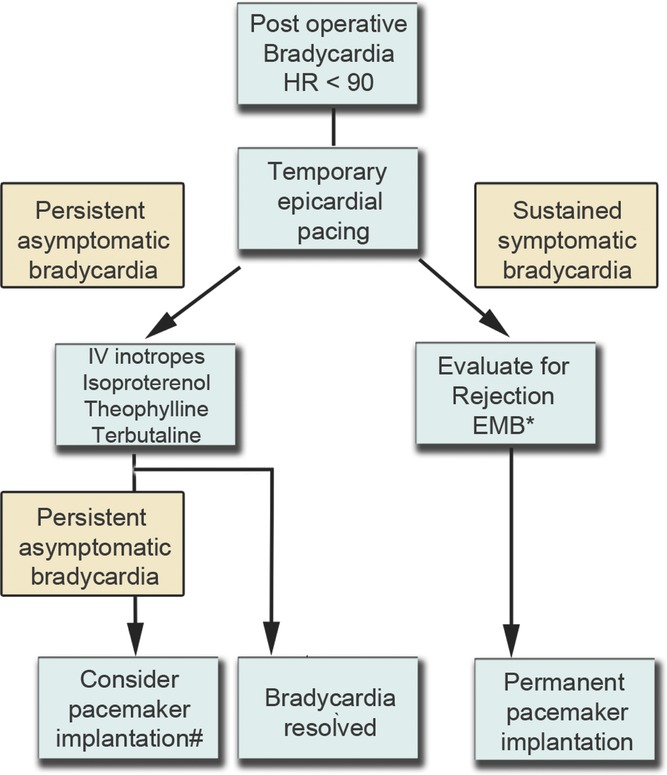
Management of postoperative bradycardia. EMB indicates endomyocardial biopsy; EPS, electrophysiology study; HR, heart rate; IV, intravenous; and PPI, permanent pacemaker implantation. ^*^Severe rejection is a relatively rare cause of bradycardia, and drug effects should be ruled out as a cause. ^#^Pacemaker is often advised for chronotropic incompetence, especially when functional rehabilitation is prevented by bradycardia.

**Figure 2.**
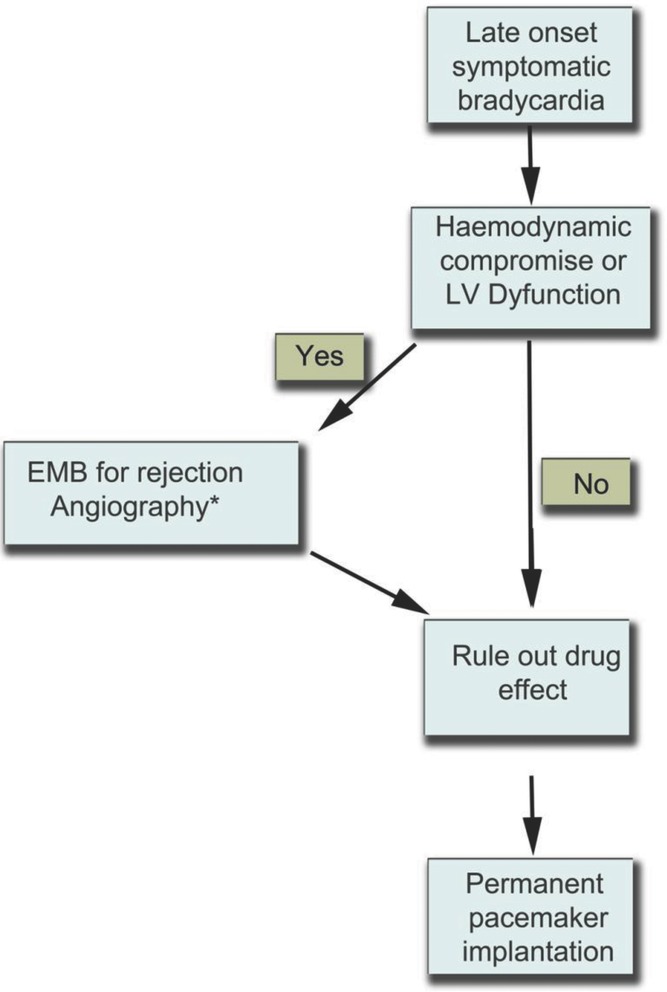
Management of symptomatic late-onset bradycardia after HT. In addition to management of bradycardia, it is imperative to manage possible rejection and significant cardiac allograft vasculopathy. ^*^All symptomatic bradycardia should be considered for biopsy and angiography.

In the perioperative period, bradycardia should be managed with temporary pacing in order to maintain heart rates higher than 90 per minute. Alternatively, isoproterenol, theophylline, or terbutaline can be used to maintain heart rate while awaiting return of normal sinus node function.^31,38^ Permanent pacemakers are generally indicated only for bradycardia that does not resolve and is associated with symptoms. Implantation of pacemaker is usually delayed until after the third week after transplantation. In most large series, permanent pacemakers were implanted in <10% of patients,^38,39^ with a few studies documenting a higher prevalence of >20%.^32,38^ In a large retrospective series, biatrial anastomosis was the major risk factor for permanent pacemaker implantation, though older donor age may contribute, and graft ischemic time has been associated in some series.^32,39,40^ Early after HT, sinus node dysfunction is the most common reason for pacemaker implantation; after 30 days, AV conduction disease and sinus node dysfunction are equally prevalent indications.^39,41^ However, biatrial anastomosis or need for permanent pacemaker implantation is not associated with decreased survival.^40,42^ A large series of patients with pacemaker implantation after HT reported that only 14.5% of patients are pacemaker dependent 6 months after implantation.^42^ Even with AV nodal disease, only 20% are pacemaker dependent.^41,42^ Usually AV block is intermittent, and permanent complete heart block is rare.^36,41^ A more recent series, however, showed that the majority of patients who had pacemakers implanted for late-onset AV block were pacemaker dependent on follow-up.^40^ A generally accepted management strategy for postoperative and late-onset bradycardia is shown in Figures 1 and 2, respectively.

Bradycardia was associated with acute rejection in a small series,^43^ but larger series do not support this observation.^28,33,40–42^ However, late complete heart block or high-grade AV block has been associated with rejection in several reports and series and is associated with worse prognosis.^34,36,43,44^ Progressive first-degree AV block with bundle branch block may portend a poor prognosis and increased risk of SCD.^45^ Pseudo AV block can be observed in cases where there is no atrial activity from the donor atria but where atrial activity in the recipient atria gives rise to the ECG appearance of nonconducted P waves.^46^

Many patients may have been maintained on amiodarone before HT. Residual effects of the drug may affect the new allograft and contribute to posttransplantation bradycardia that may persist for several weeks.

Incomplete and complete right bundle branch block are common ECG findings in various retrospective series^35,47,48^; a recent retrospective series showed a lower incidence of right bundle branch block of 20% and no association with mortality rate.^48^ However, progressive bundle branch blocks on serial ECGs were associated with increased risk of mortality and SCD.^35,49^

### Atrial Arrhythmias

The incidence of atrial arrhythmias after HT ranges between 0.3% and 24% for AF and 2.8% and 30% for atrial flutter.^14,16,24,29^ Most large series consistently noted a smaller incidence of AF and atrial flutter.^14,15,25,29^ Other arrhythmias, such as atrial tachycardia, AV nodal reentrant tachycardia, and AV reentrant tachycardia, have also been reported. AF is the most common early arrhythmia, whereas atrial flutter or macroreentrant atrial tachycardia is more common late after HT.^15,16,24,26,50^

### Atrial Fibrillation

**Figure 3.**
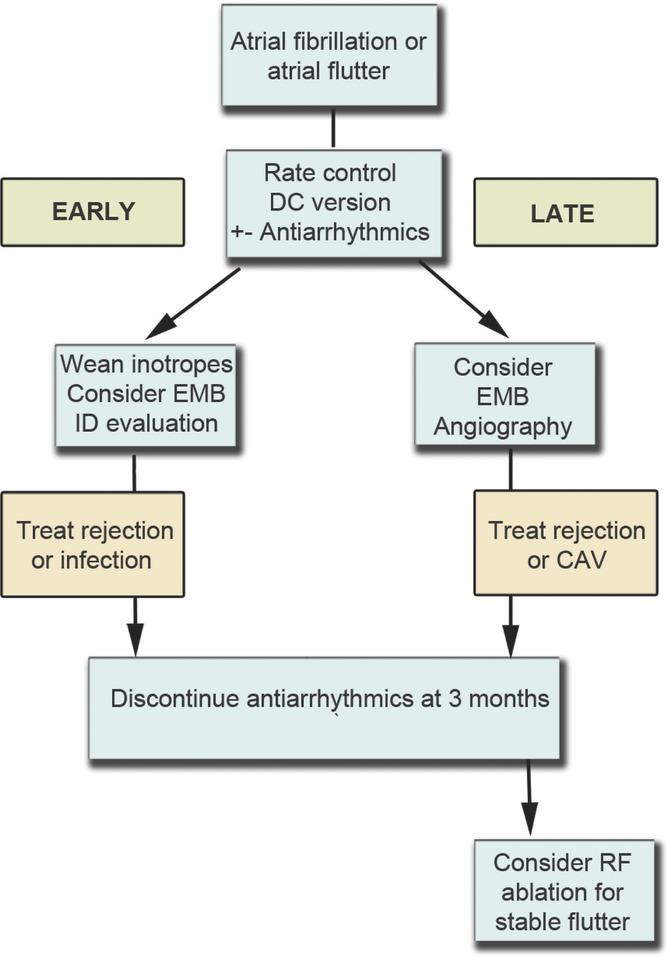
Management of early or late atrial arrhythmia after HT. CAV indicates cardiac allograft vasculopathy; EMB, endomyocardial biopsy; ID, infectious disease; and RF, radiofrequency ablation. Although rejection may underlie some cases of early AF, late AF or flutter is associated with rejection, significant graft vasculopathy, or secondary causes.

In the immediate postoperative period, many factors predispose to AF, and a high incidence has been reported.^26^ The mechanism of AF after HT is similar to other settings of postoperative AF—manipulation of the heart, pericardial inflammation, use of inotropes, and the autonomic changes after the surgery. However, AF within 2 weeks of surgery can also be associated with rejection.^14,24^ Interestingly, however, recent series show a much lower incidence of AF after HT compared to coronary artery bypass grafting, valve surgery, or even bilateral lung transplantation.^14–16^ In addition to the major mechanisms discussed above, the reduced incidence may also be due to the healthier donor heart compared to patients with chronic cardiac ischemia/infarction, severe valve disease, or severe lung disease. Moreover, although >75% of postoperative AF occurs within 7 days in the case of major thoracic surgery, only 50% of postoperative AF after HT occurs within the first 2 weeks. The management strategy for early atrial arrhythmias is similar to other settings. Figure 3 shows a general approach followed by transplant physicians for early and late arrhythmias. Late or persistent atrial arrhythmias should prompt evaluation for rejection^31^ or vasculopathy because AF is otherwise a rare occurrence in the stable transplant patient.^15^

Most HT series report that after treatment of the initial episode of AF, the majority of patients are free of recurrent AF. Thus, prolonged antiarrhythmic drug therapy is generally not indicated. Standard antiarrhythmic drugs include amiodarone and less commonly procainamide and flecainide. Antiarrhythmic agents are rarely prescribed for >3 months and the choice is narrow, especially because of increased risk of drug interactions in the heart transplant patient. Amiodarone may be used after HT but is associated with significant drug interaction with cyclosporine or tacrolimus, requiring close monitoring of immunosuppressant levels, which can become elevated. Because of amiodarone's long half-life, close monitoring of immunosuppressant levels is also required for several weeks after discontinuation of the antiarrhythmic drug. As in other situations, amiodarone is not a preferred drug for long-term use because of its side-effect profile.^51^ Dronaderone, because of its significant interaction with the calcinuerin inhibitors, is generally contraindicated in this setting. Rate control can be achieved with β-blockers and calcium channel blockers, but these should be used with caution because of risk of bradycardia and interaction with immunosuppressants, respectively. Adenosine should be administered only if strongly indicated and at low dose (3 mg, unless the patient has indwelling pacing leads) because of the risk of significant sustained bradycardia or asystole.^52^ Warfarin can either increase or decrease cyclosporine levels, and monitoring of both prothrombin time and cyclosporine levels is required on a more frequent basis. Anticoagulation in the early phase after HT is problematic because of the need to perform frequent endomyocardial biopsies. Patients may be maintained on subcutaneous low-molecular-weight heparin during this period. The recent availability of direct thrombin inhibitors (eg, dabigatran) may represent a more feasible alternative because of rapid onset of action.

AF after HT, especially when occurring >30 days postoperatively is a marker of higher long-term mortality rate.^26,27,44^ For this reason and because of the relative rarity of AF after HT, late AF occurrence should prompt evaluation for LV dysfunction, cardiac allograft vasculopathy, or acute rejection,^15,16^ as outlined in Figure 3.

### Atrial Flutter

Atrial flutter is the most common sustained atrial arrhythmia late after HT, and beyond 3 weeks of surgery, atrial flutter episodes outnumber AF.^15,16,24,29,44^ Atrial flutter is the most common arrhythmia associated with rejection.^24,44,50^ Late-onset flutter or reentrant tachycardia may reflect remodeling of the atria.^15,25^ Typical isthmus-dependent flutters may be easily identified, but atypical macroreentrant tachycardia may be difficult to differentiate from ectopic atrial tachycardia in the donor heart or atrial activity conducted from the recipient atrium when the biatrial method is used.^53,54^ Risk of atrial flutter is increased by use of the biatrial method and older donor age.^27^

Patients with atrial flutter are more likely to have LV dysfunction and earlier mortality.^24,25^ When sustained atrial flutter occurs after HT, radiofrequency ablation is a viable option after primary etiologies such as acute rejection, LV dysfunction, and cardiac ischemia have been excluded. The altered anatomy of the transplanted heart often makes catheter placement challenging for ablation of right atrial isthmus flutter.^53,55^ Mitral isthmus flutter can be ablated using standard techniques used in the non-HT context.

### Ventricular Arrhythmias and SCD

Nonsustained ventricular tachycardia (VT) can be relatively common in the early postoperative period, and possible associations with rejection or early graft failure have been variable.^28,50^ However, symptomatic nonsustained VT occurring late after HT can be associated with severe cardiac allograft vasculopathy and may warrant ICD placement.^56^ Sustained VT is infrequent after HT and when observed should prompt both coronary angiography and cardiac biopsy.^31^ Sustained VT in the immediate postoperative period may indicate impending hyperacute rejection.

The mortality rate in the later years after HT is ≈4% per year, substantially higher than the age-matched general population.^5,57^ Many die because of noncardiac causes such as malignancy. Most deaths after HT, however, are attributed to severe cardiac allograft vasculopathy and ventricular dysfunction. Approximately 25% of such patients may suffer SCD. The reported incidence varies depending on the nature of the study, whether hospital based or autopsy based, and how SCD was defined.^21,57,58^ The proximate causes for sudden death include acute ischemia, rejection, and severe LV dysfunction. Primary arrhythmic death is diagnosed when no attributable anatomic cause is identified and is described in around 25% of sudden deaths after HT.^21,58^

### Mechanisms of SCD

In a large retrospective analysis, the first recorded terminal rhythms in patients with SCD were asystole in 34%, PEA in 20%, and VF in only 10%.^21^(The other 36% had no rhythm recorded or documented during the terminal event.) Acute ischemia was identified as the cause of SCD in the majority of cases. In patients with acute ischemia dying suddenly, asystole was observed in 50%, PEA in 44%, and VF in only 6% of cases.^21^ Lack of sympathetic innervation is a possible explanation for the low rates of VF in SCD after transplantation.

### Recognition of High-Risk Individuals

Heart transplant patients with significant ventricular dysfunction and cardiac allograft vasculopathy have the highest risk of SCD.^21,56,59^ Less commonly, episodes of acute cellular rejection lead to LV dysfunction and have also been identified as an important cause of SCD, probably contributed to by the less-vigilant surveillance for rejection late after HT.^60–62^ Those with progressive conduction system disease and bundle branch block have also been reported to have higher risk of SCD. Other risk factors noted in smaller studies include frequent rejection,^24^ older donor age,^1^ myocardial hypertrophy,^1,58^ and abnormally prolonged corrected QT interval in the donor heart.^19^ Patients with a history of syncope are also at higher risk and merit electrophysiological study, coronary angiography, and possible biopsy. Notwithstanding the current guidelines that recommend pacemaker for syncope after HT, such patients may be at risk of serious ventricular arrhythmia (see below). If detailed evaluation identifies no reversible cause, ICD implantation may be considered because of the possibility of SCD, although this is not clearly supported by evidence.^3,58,63^

### Prevention

The prevention of mortality and SCD in post-HT patients revolves around prevention of progressive cardiac allograft vasculopathy and early detection and treatment of rejection. Although there are no clear guidelines on ICD implantation, some clinical observation data are available.^3,56,63^ The most common situations in which ICDs are implanted include cardiac allograft vasculopathy with LV dysfunction, nonspecific graft failure, unexplained syncope, and high nonsustained ventricular arrhythmia burden. The role of the electrophysiology study in the presence of syncope is also not clear.^56,63^ There are reports of SCD in patients with an ICD, which were presumably due to PEA because no VT or VF was documented at the time of death.^64^ There are also concerns that there is increased risk of infections and lead-related complications in such patients. However, a multicenter registry of posttransplantation patients implanted with ICDs showed that nearly one third of the patients had appropriate ICD therapy, almost all of them having significant cardiac allograft vasculopathy. Syncope or previous cardiac arrest did not predict increased risk of appropriate therapy.^63^ Therefore, multicenter studies with prolonged follow-up are warranted that will evaluate the exact role of ICDs for the long term. In our practice, we use ICDs for SCD prevention among HT patients in several contexts: (1) patients who meet conventional criteria for SCD (ejection fraction <35% or survival after prior SCD), (2) patients with sustained VT, (3) patients with frequent nonsustained VT with significant graft vasculopathy, (4) patients with syncope with inducible VT at time of electrophysiology study, and (5) patients with syncope without reversible cause or strong evidence for bradycardia as etiology (especially with LV dysfunction or significant graft vasculopathy).

## Conclusion

As survival continues to improve after HT, enhanced management of posttransplantation arrhythmias has become important for reduction of morbidity and to improve quality of life. Arrhythmias can also serve as markers of otherwise unrecognized pathologies in the transplanted heart. Therapeutic electrophysiological procedures such as pacemaker implantation and radiofrequency ablation can be effective. With better postoperative care and reduction in number of rejection episodes, the incidence of some arrhythmias such as AF has decreased. However, arrhythmias in late posttransplantation follow-up are associated with worse outcomes due to acute rejection, LV dysfunction, and SCD. From retrospective studies, the mode of SCD manifestation in the vast majority of HT patients appears to be PEA as opposed to VF. At the same time, registry data suggest that appropriate shocks were received by nearly 30% of HT patients implanted with ICDs for various indications. Given the predominance of PEA over VF as a mode of presentation, prospective evaluation is necessary to determine whether there is any role for ICDs for SCD prevention in HT patients.
